# Ordinal analysis and the set existence property for intuitionistic set theories

**DOI:** 10.1098/rsta.2022.0019

**Published:** 2023-05-29

**Authors:** Michael Rathjen

**Affiliations:** Department of Pure Mathematics, University of Leeds, Leeds LS2 9JT, UK

**Keywords:** existence property, intuitionistic set theory, ordinal analysis, realizability

## Abstract

On account of being governed by constructive logic, intuitionistic theories T often enjoy various existence properties. The most common is the *numerical existence property* (NEP). It entails that an existential theorem of T of the form (∃x∈N)A(x) can be witnessed by a numeral $\bar{n}$ such that T proves $A(\bar{n})$. While NEP holds almost universally for natural intuitionistic set theories, the general existence property (EP), i.e. the property of a theory that for every existential theorem, a provably definable witness can be found, is known to fail for some prominent intuitionistic set theories such as Intuitionistic Zermelo–Fraenkel set theory (IZF) and constructive Zermelo–Fraenkel set theory (CZF). Both of these theories are formalized with collection rather than replacement as the latter is often difficult to apply in an intuitionistic context because of the uniqueness requirement. In light of this, one is clearly tempted to single out collection as the culprit that stymies the EP in such theories. Beeson stated the following open problem: *‘Does any reasonable set theory with collection have the existence property?* and added in proof: *The problem is still open for IZF with only bounded separation*.’ (Beeson. 1985 *Foundations of constructive mathematics*, p. 203. Berlin, Germany: Springer.) In this article, it is shown that IZF with bounded separation, that is, separation for formulas in which only bounded quantifiers of the forms (∀x∈a),(∃x∈a),(∀x⊆a),(∃x⊆a) are allowed, indeed has the EP. Moreover, it is also shown that CZF with the exponentiation axiom in place of the subset collection axiom has the EP. Crucially, in both cases, the proof involves a detour through ordinal analyses of infinitary systems of intuitionistic set theory, i.e. advanced techniques from proof theory.

This article is part of the theme issue ‘Modern perspectives in Proof Theory’.

## 1. Introduction

Intuitionistic theories are renowned for very desirable meta-mathematical properties, most prominently disjunction and existence properties. There are standard tools available to obtain these properties for Heyting arithmetic and theories with quantification over sets of natural numbers or the functions from Baire space (e.g. second-order arithmetic and function arithmetic), whereas set theories with their transfinite hierarchies of sets can raise formidable technical challenges.

**Definition 1.1.**

We will consider theories T the language of which, L(T) comprises the language of the set theory. Furthermore, to simplify matters, we shall assume that L(T) has a constant ω naming the set of von Neumann natural numbers, and for each n, there is a constant $\bar{n}$ denoting the nth element of ω.

(i) T has the *disjunction property* (DP), if whenever T⊢A∨B holds for sentences A and B of T, then T⊢A or T⊢B.
(ii) T has the *numerical existence property* (NEP), if whenever T⊢(∃x∈ω)A(x) holds for a formula A(x) with at most the free variable x, then $T⊢A(\bar{n})$ for some n.
(iii) T has the *existence property* (EP), if whenever T⊢∃xA(x) holds for a formula A(x) having at most the free variable x, then there is a formula C(x) with exactly x free, so that
  T⊢∃!x [C(x)∧A(x)].

While the DP and NEP hold for many intuitionistic set theories (even with choice principles) (see [1,–6]), verifying the EP poses considerably more difficult technical problems, provided the property holds at all. As Beeson wrote in his book [1, p. 202]:

*‘It has turned out to be difficult to establish the existence property for constructive set theories, and new techniques have been developed for the purpose. From this perspective, the methods of this chapter [Chapter IX] [⋯.] represent the frontier of knowledge in the subject of constructive set theory.’*

The history of the methods that Beeson is referring to has its roots in notions of realizability developed by Kleene. Friedman [2] developed realizability notions reminiscent of Kleene’s slash [7,8]. This tool was then extended and deployed to various intuitionistic set theories by Friedman and Myhill [3,4]. Myhill [3] showed that intuitionistic ZF, when based on replacement rather than collection (notated by IZF${\;}_{R}$ from now on), has the EP. Myhill [4] did not answer the question whether the full EP still holds when one adds relativized dependent choice (RDC). Friedman & Ščedrov [9] subsequently showed this to be the case, i.e. IZF${\;}_{R}+$RDC also has the EP. Alas, none of the foregoing methods seemed to provide a tool to settle the question for set theories with collection. Then Friedman & Ščedrov [10] proved that Intuitionistic Zermelo–Fraenkel set theory (IZF) actually does not have the EP, and much later, Swan [11] showed, via a method entirely different from [10], that CZF also lacks the EP. Beeson in [1, p. 2003] stated the following open problem:

*‘Does any reasonable set theory with collection have the existence property?’* and added in proof: *‘The problem is still open for IZF with only bounded separation.’* ([1, p. 203]).

In this article, we describe a route that shows that *Intuitionistic Zermelo–Fraenkel Set Theory* with strong collection but bounded separation, i.e. separation for formulas in which only bounded quantifiers of the forms (∀x∈a),(∃x∈a),(∀x⊆a) and (∃x⊆a) are allowed, indeed has the EP. The latter theory is dubbed CZF${\;}_{P}$ in this article. Moreover, it is also shown that *constructive Zermelo–Fraenkel set theory* with the exponentiation axiom in lieu of the subset collection axiom, CZF${\;}_{E}$, has the EP. The novel technical tool featuring in this article is the employment of ordinal analysis from the proof theory that allows one, in conjunction with the reductions established in [12], to overcome the difficulties posed by collection.

A little signposting as to the contents of the article is in order. Section 2 features the *weak existence property* (wEP), which was researched in [12], and we review the main results of [12], notably that ${\mathrm{CZF}}_{P}$ and ${\mathrm{CZF}}_{E}$ possess the wEP. Moreover, [12] showed that these theories are partially conservative over their intuitionistic Kripke–Platek-like counterparts IKP(P) and IKP(E), respectively, and, moreover, that if they possess the EP for certain syntactically restricted classes of formulae, then the EP would hold for ${\mathrm{CZF}}_{P}$ and ${\mathrm{CZF}}_{E}$ tout court. This material will be reviewed in §2. Section 3 is devoted to a sketch of the ordinal analyses of IKP(P) and IKP(E). A crucial step in overcoming the problems with collection is the technique of collapsing infinite derivations whereby all instances of collection in the derivation are removed. Section 4 looks at the systems ${\mathrm{CZF}}_{P,R}$ and ${\mathrm{CZF}}_{E,R}$ that arise from ${\mathrm{CZF}}_{P}$ and ${\mathrm{CZF}}_{E}$, respectively, by replacing the strong collection scheme by the replacement scheme. With the help of the ordinal analyses from §3, it is shown that ${\mathrm{CZF}}_{P}$ and ${\mathrm{CZF}}_{E}$ are conservative over their counterparts ${\mathrm{CZF}}_{P,R}$ and ${\mathrm{CZF}}_{E,R}$ for $\Sigma^{P}$ and $\Sigma^{E}$ formulae, respectively. Section 5 is concerned with demonstrating that ${\mathrm{CZF}}_{P,R}$ and ${\mathrm{CZF}}_{E,R}$ have the EP, using well-established technology. The final §6 then reaps the fruits of all the hard work, ascertaining that ${\mathrm{CZF}}_{P}$ and ${\mathrm{CZF}}_{E}$ have the EP, too.

## 2. Preparations: from the weak to the strong existence property

When investigating the EP problem, one is naturally drawn to investigate a related but weaker form, termed the weak existence property (wEP), defined in [12] by the relaxed requirement of finding, for every existential theorem, an inhabited and provably definable set of witnesses.

**Definition 2.1. ([12, Definition 1.2])**

Let T be a theory whose language, L(T), comprises the standard language of set theory.

(i) T is said to have the wEP if whenever
  T⊢∃xA(x),
  holds for a formula A(x) having at most the free variable x, then there is a formula C(x) with exactly x free, such that
  $$\begin{array}{rl} & T⊢\exists !x\;C(x),\\ & T⊢\forall x\;[C(x)\to \exists u\;u\in x]\\ \mathrm{and}\;\;\;\; & T⊢\forall x\;[C(x)\to \forall u\in x\;A(u)]. \end{array}$$
(ii) A more general version of wEP allows for additional parameters. The *uniform weak existence property* (uwEP) is the following property: if
  T⊢ ∀u ∃xA(u,x)
  holds for a formula A(u,x) having at most the free variables u,x, then there is a formula C(u,x) with exactly u,x free, such that
  $$\begin{array}{rl} & T⊢\forall u\;\exists !x\;C(u,x),\\ & T⊢\forall u\;\forall x\;[C(u,x)\to \exists z\;z\in x],\\ \mathrm{and}\;\;\;\; & T⊢\forall u\;\forall x\;[C(u,x)\to \forall z\in x\;A(u,z)]. \end{array}$$

Clearly, uwEP subsumes wEP. As already pointed out in [12, proposition 1.3], IZF does not satisfy wEP either.

**Proposition 2.2.**

*IZF does not have the weak existence property.*

**Proof.**

According to ([10], theorem 1.1), IZF does not have the EP for some sentences of the form

2.1 ∃x [∃y D(y)→∃y∈x D(y)].

Let E(x) be the part in square brackets of the previous formula. If wEP held for IZF, then one could find a formula C(u) such that IZF⊢∃!uC(u)∧∀u [C(u)→u inhabited] and IZF⊢∀u [C(u)→∀x∈u E(x)]. But then IZF⊢∃!u[C(u)∧ E(⋃u)], contradicting the aforementioned theorem from [10].

Note that formulae of the form (2.1) are readily deducible in IZF, using Collection and Separation. Thus, in light of Myhill’s result that ${\mathrm{IZF}}_{R}$ has the EP, clearly collection is implicated in the failure of EP for IZF. It will turn out, however, that separation for formulae with unrestricted quantifiers is also responsible for this failure.

There is another important intuitionistic set theory for which EP fails. Constructive Zermelo–Fraenkel set theory (CZF) was singled out by Aczel as a theory distinguished by the fact that it has canonical interpretation in Martin–Löf type theory (cf. [13]). While Myhill isolated the *Exponentiation Axiom* as the ‘correct’ constructive counterpart of the Power Set Axiom, CZF has an axiom scheme called Subset Collection (cf. [13–15]), which is stronger than exponentiation.^1^ Subset collection implies exponentiation and is a consequence of powerset. In the presence of the other axioms of CZF, subset collection is equivalent to the *fullness* axiom (cf. [15, 5.1.2]). The latter stipulates that given any sets A and B, there exists a set C (called *full*) of multi-valued functions from A to B such that for every multi-valued function R from A to B, there exists S⊆R with S∈C.^2^ Strength-wise, an enormous hiatus separates Exponentiation from Powerset. The fullness axiom simply decrees the existence of a full set. As there does not appear to exist a method to define a full set of multi-valued functions without invoking Powerset or choice (e.g. from NN to N), the current author conjectured that CZF is another example of a set theory lacking the wEP. This was later ascertained by Andrew Swan via an ingenious combination of three realizability models.

**Theorem 2.3. (Swan)**

CZF *does not have the weak existence property*.

**Proof.**

Swan, in [11], proves that CZF fails to have the EP. His counter-example is provided by the statement that there exists a full set of multi-valued functions from N to N. However, if CZF could prove that there exists a definable inhabited set D consisting entirely of full sets of multi-valued functions from N to N, then ⋃D would furnish a provably definable set of such multi-valued functions. Accordingly, CZF lacks the wEP, too.

Clearly, wEP is an interesting property. The article [12] established that, as far as CZF is concerned, subset collection is the sole culprit for the failure of wEP. In it, several other important intuitionistic set theories with collection were shown to have the wEP, notably CZF based on exponentiation (rather than subset collection) as well as the strengthening of CZF with the powerset axiom.^3^ These theories will be delineated in detail in the next subsection.

### (a) The systems ${\mathrm{CZF}}_{E}$ and ${\mathrm{CZF}}_{P}$

CZF formulated with exponentiation, ${\mathrm{CZF}}_{E}$, has the same language as that of the classical Zermelo–Fraenkel set theory, the only non-logical symbol being ∈. Its logic is intuitionistic first-order logic with equality. Its non-logical axioms include *extensionality*, *pairing* and *union* in their usual forms. The other axioms are the following.

*Infinity:* ∃x∀u[u∈x↔(∅=u ∨ ∃v∈x u=v+1)] where v+1=v∪{v}.

*Set induction:* ∀x[∀y∈xC(y)→C(x)]→∀xC(x)

*Bounded separation:* ∀a∃b∀x[x∈b↔x∈a∧C(x)]

for all *bounded* formulae C. A set-theoretic formula is called *bounded* or *restricted* or $\Delta_{0}$ if its build-up from prime formulae uses only ¬,∧,∨,→,∀x∈y and ∃x∈y.

*Strong collection:* for all formulae C,

∀a[∀x∈a∃yC(x,y)→∃b [∀x∈a ∃y∈b C(x,y)∧∀y∈b ∃x∈a C(x,y)]].

*Exponentiation:* letting Fun(f,a,b) be short for the formula saying that f is a function from the set a to the set b, this is

∀a∀b∃c ∀f (Fun(f,a,b)→f∈c).

For the much stronger system with the powerset axiom (Pow), added, i.e.

∀x ∃y ∀z (z⊆y→z∈y),

we use the acronym ${\mathrm{CZF}}_{P}$. Observe that both exponentiation and subset collection are implied by Pow (cf. [14, proposition 7.2]).

### (b) Intuitionistic Kripke–Platek-like set theories

The proof theory of intuitionistic Kripke–Platek set theory (KP) and its beefed up versions via exponentiation and powerset, respectively, are a central tool for proving the results of this article. Recall (cf. [18]) that the axioms of the classical KP consist of *extensionality*, *pair*, *union*, *infinity and bounded separation* ∃x ∀u[u∈x↔(u∈a∧A(u))] for all bounded formulae A(u), bounded collection ∀x∈a ∃y B(x,y)→∃z ∀x∈a ∃y∈z B(x,y) for all bounded formulae B(x,y) and *set induction* ∀x [(∀y∈x C(y))→C(x)]→∀x C(x) for all formulae C(x).

By IKP, we notate the intuitionistic version of KP. It will be important later to consider a version of IKP that uses Σ replacement in lieu of $\Delta_{0}$ collection. Σ replacement is the schema

2.2 ∀x∈a ∃!y C(x,y)→∃b∀y [y∈b↔∃x∈a C(x,y)],

where C(x,y) is a Σ formula, i.e. a formula belonging to the smallest class of formulae containing the $\Delta_{0}$-formulae closed under ∧,∨ and the quantifiers ∀u∈c, ∃u∈c, ∃v. By ${\mathrm{IKP}}_{R}$, we denote the variant of IKP based on Σ replacement instead of $\Delta_{0}$ collection. Since Σ replacement is provable in IKP (e.g. [14], theorem 11.7), ${\mathrm{IKP}}_{R}$ is a subtheory of IKP.

#### i Power and exponentiation Kripke–Platek set theory

These theories come with a germane notion of bounded quantifier. Subset bounded quantifiers ∃x⊆y… and ∀x⊆y… are abbreviations for ∃x(x⊆y ∧ …) and ∀x(x⊆y→…), respectively.

A formula is said to be in $\Delta_{0}^{P}$ if all its quantifiers are of the form Q x⊆y or Q x∈y, where Q is ∀ or ∃ and x and y are distinct variables.

Let Fun(f,x,y) be an acronym for the bounded formula expressing that f is a function with domain x and co-domain y. Exponentiation bounded quantifiers ∃f∈xy… and ∀f∈xy… serve as abbreviations for ∃f(Fun(f,x,y) ∧ …) and ∀x(Fun(f,x,y)→…), respectively.

**Definition 2.4.**

The class of $\Delta_{0}^{P}$ contains the atomic formulae and is closed under ∧,∨,→,¬ and the quantifiers

∀x∈a, ∃x∈a, ∀x⊆a, ∃x⊆a.

The class of $\Delta_{0}^{E}$ formulae contains the atomic formulae and is closed under ∧,∨,→,¬ and the quantifiers

∀x∈a, ∃x∈a, ∀f∈ab, ∃f∈ab.

**Definition 2.5.**

IKP(E) has the same language and logic as IKP. Its axioms are extensionality, pairing, union, infinity, exponentiation, $\Delta_{0}^{E}$-separation and $\Delta_{0}^{E}$-collection.

IKP(P) has the same language and logic as IKP. Its axioms are extensionality, pairing, union, infinity, powerset, $\Delta_{0}^{P}$-separation and $\Delta_{0}^{P}$-collection.

The transitive classical models of IKP(P) are called power admissible sets in [19].

An alternative way of axiomatizing IKP(P) proceeds by adding a function symbol P for the powerset function as a primitive symbol to the language and the axiom

∀y [y∈P(x)↔y⊆x],

and allowing the new symbol to occur in the schemes of $\Delta_{0}$ separation and collection to the $\Delta_{0}$ formulae of this new language. Similarly, IKP(E) can be formulated by adding a primitive function symbol E for the exponentiation function.

**Lemma 2.6. ([12, lemma 2.4])**

*Let* ${\mathrm{CZF}}^{-}$ *be* CZF *without subset collection. The following hold:*

(i) IKP *is a subtheory of* ${\mathrm{CZF}}^{-}$;
(ii) IKP(E) *is a subtheory of* ${\mathrm{CZF}}_{E}$;
(iii) IKP(P) *is a subtheory of* ${\mathrm{CZF}}_{P}$.

Note that the foregoing Lemma implies that, despite having just $\Delta_{0}$-separation at the axiomatic level, ${\mathrm{CZF}}_{E}$ proves $\Delta_{0}^{E}$-separation and ${\mathrm{CZF}}_{P}$ proves $\Delta_{0}^{P}$-separation.

### (c) Conservativity over intuitionistic Kripke–Platek set theories

As shown in [12], ${\mathrm{CZF}}^{-}$, ${\mathrm{CZF}}_{E}$ and ${\mathrm{CZF}}_{P}$ are conservative over their intuitionistic Kripke–Platek counterparts for syntactically restricted forms of formulae. These results will be of relevance later.

The Σ formulae constitute an important syntactic class in the KP (cf. [18]). Their extensions to the contexts of IKP(E) and IKP(P) (singled out in [12], definition 2.5) are equally important.

**Definition 2.7.**

The Σ formulae are the smallest class of formulae containing the $\Delta_{0}$-formulae closed under ∧,∨ and the quantifiers ∀x∈a,∃x∈a,∃x.

The $\Sigma^{E}$ formulae are the smallest class of formulae containing the $\Delta_{0}^{E}$-formulae closed under ∧,∨ and the quantifiers ∀x∈a,∃x∈a,∀f∈ab,∃f∈ab,∃x.

The $\Sigma^{P}$ formulae are the smallest class of formulae containing the $\Delta_{0}^{P}$-formulae closed under ∧,∨ and the quantifiers ∀x∈a,∃x∈a,∀x⊆a,∃x⊆a,∃x.

**Definition 2.8.**

We say that a formula D is $\Pi_{2}$, $\Pi_{2}^{E}$ or $\Pi_{2}^{P}$ if it is of the form ∀x ∃y A(x,y) with A(x,y) being, respectively, $\Delta_{0}$, $\Delta_{0}^{E}$ and $\Delta_{0}^{P}$.

**Theorem 2.9. ([12, theorem 4.8])**

(i) ${\mathrm{CZF}}^{-}$ *is conservative over* IKP *for* $\Pi_{2}$ *sentences*.
(ii) ${\mathrm{CZF}}_{E}$ *is conservative over* IKP(E) *for* $\Pi_{2}^{E}$ *sentences*.
(iii) ${\mathrm{CZF}}_{P}$ *is conservative over* IKP(P) *for* $\Pi_{2}^{P}$ *sentences*.

### (d) Towards the existence property

As it will turn out, a first crucial step towards establishing the EP was already achieved in [12]. To see this, though, it is fruitful to scour the details of the proofs leading to the following result.

**Theorem 2.10. ([12, theorem 3.10])**

IKP, IKP(E), IKP(P), ${\mathrm{CZF}}^{-}$, ${\mathrm{CZF}}_{E}$ *and* ${\mathrm{CZF}}_{P}$ *have the wEP. Indeed, they satisfy the stronger property* uwEP.^4^

A few comments as to the technology deployed in [12] are in order. The main tool consists of a notion of realizability where realizers are programs for various notions of set recursive functions (such functions were e.g. studied in [20–23]), especially power and exponentiation recursive functions. Moreover, realizers for existential formulas consist of inhabited sets of realizers, and the notion of realizability is paired with that of truth. This notion is completely different from the generic realizability used by Beeson ([1], VIII.6) and McCarty [24] that has its roots in a notion of realizability for intuitionistic second-order arithmetic due to Kreisel & Troelstra [25]. The idea of using sets of realizers rather than single ones is akin to the Diller & Nahm [26,27] variant of Gödel’s functional interpretation.

Let T be any of the theories ${\mathrm{CZF}}^{-}$, ${\mathrm{CZF}}_{E}$ or ${\mathrm{CZF}}_{P}$. According to [12], the goal of settling the EP for T can be achieved by tackling the perhaps more manageable question of whether the Kripke–Platek counterpart of T has the EP for Σ, $\Sigma^{P}$ and $\Sigma^{E}$ existential theorems, respectively.

**Definition 2.11.**

Let Ξ be a set of formulae. T is said to have the EP for Ξ if whenever T⊢∃xA(x) for a sentence ∃xA(x) with A(x) in Ξ, then one finds a formula C(x) (with at most x free) such that

T⊢∃!x [C(x) ∧ A(x)].

In [12, definition 5.1], it was also required that C(x) belongs to Ξ. But this is not really necessary for the applications made in [12, theorems 5.2 and 5.3], and hence, we use the relaxed notion.

**Theorem 2.12. ([12, theorems 5.2, 5.3])**

(i) *If* IKP *has the* EP *for* Σ *formulae, then* IKP *and* ${\mathrm{CZF}}^{-}$ *have the* EP.
(ii) If IKP(E) has the EP for $\Sigma^{E}$ formulae, then IKP(E) and ${\mathrm{CZF}}_{E}$ have the EP.
(iii) If IKP(P) has the EP for $\Sigma^{P}$ formulae, then IKP(P) and ${\mathrm{CZF}}_{P}$ have the EP.

In the cases of IKP and ${\mathrm{CZF}}^{-}$, it has already been shown that the strategy suggested by theorem 2.12 can be successful. Theorem 6.1 will furnish yet another proof.

**Corollary 2.13.**

IKP *and* ${\mathrm{CZF}}^{-}$ *have the* EP.

**Proof.**

This result is stated as Corollary 6.1 in [12]. There is a sketch of a proof in [12], anticipating the ordinal analysis of IKP from [28]. More details are provided in the proofs of [28, theorem 2.35 and remark 2.36]. There it is shown that if IKP⊢∃x C(x) for a Σ-sentence, then there is a cut-free proof of it in the infinitary proof system ${\mathrm{IRS}}_{\Omega }$, which can then be milked to extract a term $\bar{s}$ of ${\mathrm{IRS}}_{\Omega }$ and an ordinal representation $\bar{\alpha }<\Omega$ in $B^{\Omega }(\varepsilon_{\Omega +1})$ such that ${\mathrm{IRS}}_{\Omega }$ proves $C{\left(\bar{s}\right)}^{L_{\bar{\alpha }}}$. The entire ordinal analysis of the infinitary proof can already be formalized in IKP. As a consequence, IKP proves that there is an ordinal α denoted by $\bar{\alpha }$ and a set s denoted by $\bar{s}$ such that $L_{\alpha }⊨C(s)$. This is then also a fact provable in ${\mathrm{CZF}}^{-}$, and thus, since ${\mathrm{CZF}}^{-}$ has the numerical EP by the proof of theorem 6.1 in [5] (just ignore the subset collection part), there are concrete such terms $\bar{\alpha },\bar{s}$ (note that terms of ${\mathrm{IRS}}_{\Omega }$ can be assumed to be coded as naturals), which can be described by Σ formulae, say $B_{1}(x)$ and $B_{2}(x)$. As a result, we have ${\mathrm{CZF}}^{-}⊢\exists !\alpha \;\exists !s\;[B_{1}(\alpha )\;\wedge \;B_{2}(s)\;\wedge \;L_{\alpha }⊨C(s)]$. The latter being a Σ formula, it is also provable in IKP by theorem 2.9 (i). Hence, IKP has the EP for Σ formulae. In the light of theorem 2.12 (i), it follows that IKP and ${\mathrm{CZF}}^{-}$ have the EP.

## 3. Ordinal analyses of Kripke–Platek-like intuitionistic set theories

According to theorem 2.12, one could ascertain the EP for ${\mathrm{CZF}}_{E}$ and ${\mathrm{CZF}}_{P}$, respectively, if one succeeded in establishing the EP for $\Sigma^{E}$ formulae for IKP(E) and the EP for $\Sigma^{P}$ formulae for IKP(P), respectively. The usual techniques for showing the EP (cf. [1], chapter IX), however, do not work for theories with collection. The ordinal-theoretic proof theory, though, has developed tools for eliminating collection axioms from proofs in infinitary proof systems, using intricate techniques for collapsing derivations. Originally, these techniques were developed for classical theories much weaker than IKP(P) [29,30]. An important step towards an ordinal analysis of the classical Power Kripke–Platek set theory was taken in [31]. A further important step was taken by Cook & Rathjen in [28], furnishing ordinal analyses of intuitionistic power and exponentiation Kripke–Platek set theory. Especially the treatment of the latter theory turned out to be wickedly complex. In the case of intuitionistic power Kripke–Platek set theory, one can base the infinitary proof system on a term structure where one can directly read off a term’s level in the power hierarchy. This is not possible in the pertinent ‘exponentiation hierarchy’. Instead, a term’s level is viewed as a changeable property within the infinitary system.

The technical source for this chapter is the long article [28] (93 pages) by Cook & Rathjen. We will adumbrate its contents, not only for the reader's sake but also in order to extract some of its hidden implications.

### (a) Ordinal analysis of IKP(P)

We first turn to IKP(P) because it is much simpler to deal with. In what follows, the proof system for IKP(P) will be the usual Gentzen sequent calculus for intuitionistic logic, which derives intuitionistic sequents of the form Γ⇒Δ, where Γ and Δ are finite sets of formulae and Δ contains at most one formula.

**Definition 3.1.**

One formal point is that while previously subset bounded quantifiers

(∀x⊆a)A(x)and(∃x⊆a)A(x)

were viewed as abbreviations, here they are treated as quantifiers in their own right, not abbreviations. Accordingly, these quantifiers require their own logical rules.

$$(pb\exists L)\frac{\Gamma ,a\subseteq b\wedge F(a)\Rightarrow \Delta }{\Gamma ,(\exists x\subseteq b)F(x)\Rightarrow \Delta }\;(pb\exists R)\frac{\Gamma \Rightarrow a\subseteq b\wedge F(a)}{\Gamma \Rightarrow (\exists x\subseteq b)F(x)}$$

$$(pb\forall L)\frac{\Gamma ,a\subseteq b\to F(a)\Rightarrow \Delta }{\Gamma ,(\forall x\subseteq b)F(x)\Rightarrow \Delta }\;(pb\forall R)\frac{\Gamma \Rightarrow a\subseteq b\to F(a)}{\Gamma \Rightarrow (\forall x\subseteq b)F(x)}$$

of course, where a is not to occur in the conclusion of the rules (pb∃L) and (pb∀R).

#### (i) An ordinal representation system

Following [28, definition 2.2], we provide a very brief description of a primitive recursive ordinal notation system for the *Bachmann–Howard ordinal*.

**Definition 3.2.**

Let Ω be a ‘big’ ordinal (in fact we could have chosen $\omega_{1}^{CK}$, see [32]). We define the sets $B^{\Omega }(\alpha )$ and ordinals $\psi_{\Omega }(\alpha )$ by transfinite recursion on α as follows:

3.1 $$B^{\Omega }(\alpha )=\left\{ \begin{array}{l} \mathrm{closure}\;\mathrm{of}\;\{0,\Omega \}\;\mathrm{under}:\\ +,\xi \mapsto \omega^{\xi }\\ (\xi ⟼\psi_{\Omega }(\xi ){)}_{\xi <\alpha } \end{array}\right.$$

and

3.2 $$\psi_{\Omega }(\alpha )=\min\{\rho <\Omega :\rho \notin B^{\Omega }(\alpha )\}.$$

As it turns out, $\psi_{\Omega }(\alpha )$ is always defined, and therefore, $\psi_{\Omega }(\alpha )<\Omega .$ Furthermore, it is the case that letting $B^{\Omega }(\alpha )\cap \Omega :=\{\alpha \in B^{\Omega }(\alpha )|\alpha <\Omega \}$, we have $B^{\Omega }(\alpha )\cap \Omega =\{\beta |\beta <\psi_{\Omega }(\alpha )\}$.

Let $\varepsilon_{\Omega +1}$ be the least ordinal η>Ω such that $\omega^{\eta }=\eta$. The set $B^{\Omega }(\varepsilon_{\Omega +1})$ engenders a primitive recursive ordinal representation system [33,34]. The ubiquitous ordinal $\psi_{\Omega }(\varepsilon_{\Omega +1})$ is known as the Bachmann–Howard ordinal. In the literature, one finds several equivalent variants of the representation system for this ordinal.

#### (ii) The infinitary system ${\mathrm{IRS}}_{\Omega }^{P}$

Next, we introduce the infinitary proof system ${\mathrm{IRS}}_{\Omega }^{P}$ from [28].

**Definition 3.3. ([28, Definition 2.3])**

All ordinals are assumed to be members of $B^{\Omega }(\varepsilon_{\Omega +1})$. When defining the ${\mathrm{IRS}}_{\Omega }^{P}$ terms, we also assign an ordinal level, ∣ t ∣.

1. For each α<Ω, $V_{\alpha }$ is an ${\mathrm{IRS}}_{\Omega }^{P}$ term with $|\;V_{\alpha }\;|=\alpha$.
2. For each α<Ω, we have infinitely many free variables $a_{0}^{\alpha },a_{1}^{\alpha },a_{2}^{\alpha },\ldots .$, with $|\;a_{i}^{\alpha }\;|=\alpha$.
3. If $F(x,\bar{y}\;)$ is a $\Delta_{0}^{P}$-formula of IKP(P) (whose free variables are exactly those indicated) and $\bar{s}\equiv s_{1},\ldots ,s_{n}$ are ${\mathrm{IRS}}_{\Omega }^{P}$ terms, then the formal expression $\left[x\in V_{\alpha }|F(x,\bar{s}\;)\right]$ is an ${\mathrm{IRS}}_{\Omega }^{P}$ term with $|\;\left[x\in V_{\alpha }|F(x,\bar{s}\;)\right]\;|:=\alpha$.

Note that ${\mathrm{IRS}}_{\Omega }^{P}$-terms can contain subterms of a higher level, or from higher up the von Neumann hierarchy in the intended interpretation. This reflects the impredicativity of the power set operation. The ${\mathrm{IRS}}_{\Omega }^{P}$ formulae are of the form $A(s_{1},\ldots ,s_{n})$, where $A(a_{1},\ldots ,a_{n})$ is a formula of IKP(P) with all free variables indicated and $s_{1},\ldots ,s_{n}$ are ${\mathrm{IRS}}_{\Omega }^{P}$ terms. A formula $A(s_{1},\ldots ,s_{n})$ of ${\mathrm{IRS}}_{\Omega }^{P}$ is $\Delta_{0}^{P}$ if $A(a_{1},\ldots ,a_{n})$ is a $\Delta_{0}^{P}$ formula of IKP(P).

The ${\dot{\Sigma }}^{P}$ formulae of ${\mathrm{IRS}}_{\Omega }^{P}$ are the smallest collection containing the $\Delta_{0}^{P}$-formulae and containing A∨B, A∧B, (∀x∈s)A, (∃x∈s)A, (∀x⊆s)A, (∃x⊆s)A, ∃xA, ¬C and C→A whenever it contains A and B and C is a ${\dot{\Pi }}^{P}$-formula. The ${\dot{\Pi }}^{P}$-formulae are the smallest collection containing the $\Delta_{0}^{P}$ formulae and containing A∨B, A∧B, (∀x∈s)A, (∃x∈s)A, (∀x⊆s)A, (∃x⊆s)A, ∀xA, ¬D and D→A whenever it contains A and B and D is a ${\dot{\Sigma }}^{P}$-formula.^5^

Following ([28], definition 3.2), the *axioms* of ${\mathrm{IRS}}_{\Omega }^{P}$ are as follows:

(A1) Γ,A⇒A for A in $\Delta_{0}^{P}$.
(A2) Γ⇒t=t.
(A3) $\Gamma ,s_{1}=t_{1},\ldots ,s_{n}=t_{n},A(s_{1},\ldots ,s_{n})\Rightarrow A(t_{1},\ldots ,t_{n})$ for $A(s_{1},\ldots ,s_{n})$ in $\Delta_{0}^{P}$.
(A4) $\Gamma \Rightarrow s\in V_{\alpha }$ if ∣ s ∣<α.
(A5) $\Gamma \Rightarrow s\subseteq V_{\alpha }$ if ∣ s ∣≤α.
(A6) $\Gamma ,t\in [x\in V_{\alpha }|F(x,\bar{s})]\Rightarrow F(t,\bar{s})$ for $F(t,\bar{s})$ is $\Delta_{0}^{P}$ and ∣ t ∣<α.
(A7) $\Gamma ,F(t,\bar{s})\Rightarrow t\in [x\in V_{\alpha }|F(x,\bar{s})]$ for $F(t,\bar{s})$ is $\Delta_{0}^{P}$ and ∣ t ∣<α.

The *inference rules* of ${\mathrm{IRS}}_{\Omega }^{P}$ are as follows:

 (b∀L)Γ, s∈t→F(s)⇒ΔΓ,(∀x∈t)F(x)I⇒Δ if ∣ s ∣<∣ t ∣ (b∀R)∞Γ⇒s∈t→F(s) for all ∣ s ∣<∣ t ∣Γ⇒(∀x∈t)F(x)I (b∃L)∞Γ,s∈t∧F(s)⇒Δ for all ∣ s ∣<∣ t ∣Γ,(∃x∈t)F(x)I⇒Δ (b∃R)Γ⇒s∈t∧F(s)Γ⇒(∃x∈t)F(x)I if ∣ s ∣<∣ t ∣ (pb∀L)Γ,s⊆t→F(s)⇒ΔΓ,(∀x⊆t)F(x)I⇒Δ if ∣ s ∣≤∣ t ∣ (pb∀R)∞Γ⇒s⊆t→F(s) for all ∣ s ∣≤∣ t ∣Γ⇒(∀x⊆t)F(x)I (pb∃L)∞Γ,s⊆t∧F(s)⇒Δ for all ∣ s ∣≤∣ t ∣Γ,(∃x⊆t)F(x)I⇒Δ (pb∃R)Γ⇒s⊆t∧F(s)Γ⇒(∃x⊆t)F(x)I if ∣ s ∣≤∣ t ∣ (∀L)Γ,F(s)⇒ΔΓ,∀x F(x)I⇒Δ

 (∀R)∞Γ⇒F(s) for all sΓ⇒∀x F(x)I (∃ L)∞Γ,F(s)⇒Δ for all sΓ,∃x F(x)I⇒Δ (∃ R)Γ⇒F(s)Γ⇒∃x F(x)I (∈L)∞Γ,r∈t∧r=s⇒Δ for all ∣ r ∣<∣ t ∣Γ,s∈tI⇒Δ

 (∈R)Γ⇒r∈t∧r=sΓ,s∈tI if ∣ r ∣<∣ t ∣ (⊆L)∞Γ,r⊆t∧r=s⇒Δ for all ∣ r ∣≤∣ t ∣Γ,s⊆tI⇒Δ (⊆R)Γ⇒r⊆t ∧ r=sΓ⇒s⊆tI  if ∣ r ∣≤∣ s ∣

(Cut) Γ,A⇒ΔΓ⇒AΓI⇒Δ (Σ˙P-Ref)Γ⇒AΓ⇒ ∃z AzII  if A is a Σ˙P-formula, 

as well as the rules (∧L), (∧R), (∨L), (∨R), (¬L), (¬R), (⊥), (→L) and (→R) from ${\mathrm{IRS}}_{\Omega }$. Here, as per usual, $A^{z}$ results from A by restricting all unbounded quantifiers to z.

**Definition 3.4.**

The *rank* of a formula is defined in ([28], definition 3.3) as follows.

(i) rk(s∈t):=max{∣ s ∣+1,∣ t ∣+1}.
(ii) $rk((\exists x\in t)F(x)):=rk((\forall x\in t)F(x)):=\max\{|\;t\;|,rk(F(V_{0}))+2\}.$
(iii) $rk((\exists x\subseteq t)F(x)):=rk((\forall x\subseteq t)F(x)):=\max\{|\;t\;|+1,rk(F(V_{0}))+2\}.$
(iv) $rk(\exists x\;F(x)):=rk(\forall x\;F(x)):=\max\{\Omega ,rk(F(V_{0}))+2\}.$
(v) rk(A∧B):=rk(A∨B):=rk(A→B):=max{rk(A),rk(B)}+1.
(vi) rk(¬A):=rk(A)+1.

*Crucially, observe that a formula with only bounded quantifiers has a rank <Ω, whereas a formula with an unbounded quantifier has a rank ≥Ω.*

We take the notion of operator controlled derivability for ${\mathrm{IRS}}_{\Omega }^{P}$ from Definition 3.6 [28] (originally due to Buchholz [35]), where the relation $H|\frac{\alpha }{\rho }\Gamma \Rightarrow \Delta$ is defined for an operator H, ordinals α,ρ and Γ⇒Δ, an intuitionistic sequent of ${\mathrm{IRS}}_{\Omega }^{P}$ formulae. We will just highlight the conditions for two crucial inferences.

$$\text{(Cut)}\;\frac{H|\frac{\alpha_{0}}{\rho }\Gamma ,B\Rightarrow \Delta \;\;\;\;H|\frac{\alpha_{0}}{\rho }\Gamma \Rightarrow B}{H|\frac{\alpha }{\rho }\Gamma \Rightarrow \Delta }\;\begin{array}{r} \alpha_{0}<\alpha \\ rk(B)<\rho \end{array}$$

and

$$({\dot{\Sigma }}^{P}\text{-}Ref)\;\frac{H|\frac{\alpha_{0}}{\rho }\Gamma \Rightarrow A}{H|\frac{\alpha }{\rho }\Gamma \Rightarrow \exists z\;A^{z}}\;\begin{array}{r} \alpha_{0}+1,\Omega <\alpha \\ A\text{ is a }{\dot{\Sigma }}^{P}-\text{formula} \end{array}$$

#### (iii) Embedding IKP(P) into ${\mathrm{IRS}}_{\Omega }^{P}$

The first important result that links IKP(P) to ${\mathrm{IRS}}_{\Omega }^{P}$ is that every deduction in IKP(P) can be transformed into one in the infinitary system ${\mathrm{IRS}}_{\Omega }^{P}$.

**Theorem 3.5. ([28, theorem 3.24])**

*If* $\mathrm{IKP}(P)⊢\Gamma (\bar{a})\Rightarrow \Delta (\bar{a})$, *where* $\Gamma (\bar{a})\Rightarrow \Delta (\bar{a})$ *is an intuitionistic sequent containing exactly the free variables* $\bar{a}=a_{1},\ldots ,a_{n}$, *then there exists an* m<ω *(which we may calculate from the derivation) such that*

$$H[\bar{s}]\frac{\Omega \cdot \omega^{m}}{\Omega +m}\Gamma (\bar{s})\Rightarrow \Delta (\bar{s}),$$

for any operator H and any ${\mathrm{IRS}}_{\Omega }^{P}$ terms $\bar{s}=s_{1},\ldots ,s_{n}$.

#### (iv) Cut elimination for ${\mathrm{IRS}}_{\Omega }^{P}$

The main advantage of ${\mathrm{IRS}}_{\Omega }^{P}$ over IKP(P) is the former’s amenability to partial cut elimination and the possibility to remove instances of $\left({\dot{\Sigma }}^{P}\text{-}Ref\right)$, which embody collection, from certain derivations via collapsing.

**Theorem 3.6. (Partial cut elimination for ${\mathrm{IRS}}_{\Omega }^{P}$)**

*[[28], theorem 3.11] If* $H|\frac{\alpha }{\Omega +n+1}\Gamma \Rightarrow \Delta$, *then* $H|\frac{\omega_{n}(\alpha )}{\Omega +1}\Gamma \Rightarrow \Delta$, *where* $\omega_{0}(\beta ):=\beta$ *and* $\omega_{k+1}(\beta ):=\omega^{\omega_{k}(\beta )}$.

**Lemma 3.7. (Boundedness)**

*[[28], lemma 3.12] If* A *is a* ${\dot{\Sigma }}^{P}$-*formula*, B *is a* ${\dot{\Pi }}^{P}$-*formula*, α≤β<Ω *and* β∈H, *then*

(i) *If* $H|\frac{\alpha }{\rho }\Gamma \Rightarrow A$ *then* $H|\frac{\alpha }{\rho }\Gamma \Rightarrow A^{V_{\beta }}$.
(ii) *If* $H|\frac{\alpha }{\rho }\Gamma ,B\Rightarrow \Delta$ *then* $H|\frac{\alpha }{\rho }\Gamma ,B^{V_{\beta }}\Rightarrow \Delta \;.$

Boundedness is a crucial tool in the next result.

**Theorem 3.8. (Collapsing for ${\mathrm{IRS}}_{\Omega }^{P}$)**

*[[28], theorem 3.13] Suppose that* $\eta \in H_{\eta }$, Δ *is a set of at most one* ${\dot{\Sigma }}^{P}$-*formula and* Γ *a set of* ${\dot{\Pi }}^{P}$-*formulae. Then*

$$H_{\eta }|\frac{\alpha }{\Omega +1}\Gamma \Rightarrow \Delta \;\text{implies}\;H_{\hat{\alpha }}|\frac{\psi_{\Omega }(\hat{\alpha })}{\psi_{\Omega }(\hat{\alpha })}\Gamma \Rightarrow \Delta .$$

*Here*, $\hat{\beta }=\eta +\omega^{\Omega +\beta }$ *and the operators* $H_{\xi }$ *are those defined in* [28] definition 2.18.

*In actuality, in light of 3.7, we have*

$$H_{\eta }|\frac{\alpha }{\Omega +1}\Gamma \Rightarrow \Delta \;\text{implies}\;H_{\hat{\alpha }}|\frac{\psi_{\Omega }(\hat{\alpha })}{\psi_{\Omega }(\hat{\alpha })}\Gamma^{V_{\tau }}\Rightarrow \Delta^{V_{\tau }},$$

*with* $\tau :=\psi_{\Omega }(\hat{\alpha })$.

Observe that the collapsing theorem eliminates all inference rules $\left({\dot{\Sigma }}^{P}\text{-}Ref\right)$ in the derivation.

### (b) Ordinal analysis of IKP(E)

In the same vein as for IKP(P), one can furnish an ordinal analysis for the Kripke–Platek system IKP(E) based on exponentiation, though this is much more difficult than for IKP(P). It was achieved in [28] via the infinitary proof system ${\mathrm{IRS}}_{\Omega }^{E}$. Again, we will briefly introduce the main features of ${\mathrm{IRS}}_{\Omega }^{E}$ and sketch the main results from [28] needed for this article.

#### (i) A sequent calculus formulation of IKP(E)

**Definition 3.9.**

In the sequent calculus rendering of IKP(E), there are additional *exponentiation bounded quantifiers* of the form

$$(\forall x\in^{a}b)A(x)\;\text{and}\;(\exists x\in^{a}b)A(x),$$

treated as quantifiers in their own right, not abbreviations. Quantifiers of the form ∀x, ∃x will be called unbounded, whereas the quantifiers $\left(\forall x\in^{a}b),(\exists x\in^{a}b),(\forall x\in a),(\exists x\in a\right)$ count as bounded ones. A $\Delta_{0}^{E}$-formula of IKP(E) is one that contains only bounded quantifiers.

As mentioned earlier, IKP(E) derives intuitionistic sequents, like in the following axiom for

*Exponentiation:* $\Gamma \Rightarrow \exists z\;(\forall x\in^{a}b)(x\in z)$.

To express the rules for the exponentiation bounded quantifiers, one uses a formula ‘fun(x,a,b)’, whose intuitive meaning is ‘x is a function from a to b’:

$$\begin{array}{rl} \text{fun}(x,a,b) & \;:=x\subseteq a\times b\wedge (\forall y\in a)(\exists z\in b)((y,z)\in x)\\ & \;\;\wedge (\forall y\in a)(\forall z_{1}\in b)(\forall z_{2}\in b)[((y,z_{1})\in x\wedge (y,z_{2})\in x)\to z_{1}=z_{2}]. \end{array}$$

The rules are as follows:

$$(\varepsilon b\exists L)\frac{\Gamma ,\mathrm{fun}(c,a,b)\wedge F(c)\Rightarrow \Delta }{\Gamma ,(\exists x\in {\;}^{a}b)F(x)\Rightarrow \Delta }\;(\varepsilon b\exists R)\frac{\Gamma \Rightarrow \mathrm{fun}(c,a,b)\wedge F(c)}{\Gamma \Rightarrow (\exists x\in {\;}^{a}b)F(x)}$$

$$(\varepsilon b\forall L)\frac{\Gamma ,\mathrm{fun}(c,a,b)\to F(c)\Rightarrow \Delta }{\Gamma ,(\forall x\in {\;}^{a}b)F(x)\Rightarrow \Delta }\;(\varepsilon b\forall R)\frac{\Gamma \Rightarrow \mathrm{fun}(c,a,b)\to F(c)}{\Gamma \Rightarrow (\forall x\in {\;}^{a}b)F(x)}$$

with the proviso that the variable c in (Eb∃L) and (Eb∀R) is an eigenvariable.

#### (ii) The infinitary system ${\mathrm{IRS}}_{\Omega }^{E}$

Next we need the infinitary system ${\mathrm{IRS}}_{\Omega }^{E}$ of [28, section 4.2], within which one can embed IKP(E) and carry out an ordinal analysis.

**Definition 3.10.**

Akin to the von Neumann hierarchy built by iterating the powerset operation, one may define an exponentiation hierarchy through the ‘ordinals’ of $B^{\Omega }(\varepsilon_{\Omega +1})\cap \Omega :=\{\alpha \in B^{\Omega }(\varepsilon_{\Omega +1})|\alpha <\Omega \}$ as follows:

$$\begin{array}{rl} & E_{0}:=\emptyset \;\text{and}\;E_{1}:=\{\emptyset \},\\ & E_{\alpha +2}:=\{X|X\text{ is definable over }\langle E_{\alpha +1},\in \rangle \text{ with parameters}\}\\ & \;\;\cup \{f\;|\;\text{fun}(f,a,b)\text{ for some }a,b\in E_{\alpha }\},\\ & E_{\lambda }:=\underset{\beta <\lambda }{⋃}E_{\beta }\;\text{for }\lambda \text{ a limit,}\\ \mathrm{and}\;\;\;\; & E_{\lambda +1}:=\{X\;|\;X\text{ is definable over }\langle E_{\alpha +1},\in \rangle \text{ with parameters}\}\;\text{for }\lambda \text{ a limit}. \end{array}$$

The sets $E_{\alpha }$ are transitive; see [28, lemma 4.2].

Note that the E-hierarchy can be defined in ${\mathrm{IKP}}_{R}(E)$ up to any ordinal representation α<Ω for which transfinite induction is provable as the case distinctions therein are decidable for ordinal representations. The idea behind ${\mathrm{IRS}}_{\Omega }^{E}$ is to serve as a proof system for reasoning about the E-hierarchy.

**Definition 3.11.**

The terms of ${\mathrm{IRS}}_{\Omega }^{E}$ are defined as follows:

1. $E_{\alpha }$ is an ${\mathrm{IRS}}_{\Omega }^{E}$ term for each α<Ω.
2. $a_{i}^{\alpha }$ is an ${\mathrm{IRS}}_{\Omega }^{E}$ term for each α<Ω and each i<ω, these terms will be known as ${\mathrm{IRS}}_{\Omega }^{E}$’s free variables.
3. If $F(a,b_{1},\ldots ,b_{n})$ is a $\Delta_{0}^{E}$ formula of IKP(E) containing exactly the free variables indicated, and $t,s_{1},\ldots ,s_{n}$ are ${\mathrm{IRS}}_{\Omega }^{E}$ terms, then
  $$\left[x\in t|F(x,\bar{s})\right]$$
  is also a term of ${\mathrm{IRS}}_{\Omega }^{E}$.

Note that while it was straightforward to assign the level α to a complex term $\left[x\in V_{\alpha }|F(x,\bar{s}\;)\right]$ of ${\mathrm{IRS}}_{\Omega }^{P}$ by viewing it as a denizen of the von Neumann hierarchy, it is not clear how to locate an ${\mathrm{IRS}}_{\Omega }^{E}$ term within the E-hierarchy just by looking at the syntactic build-up of that term.

The formulae of ${\mathrm{IRS}}_{\Omega }^{E}$ are of the form $F(s_{1},\ldots ,s_{n})$, where $F(a_{1},\ldots ,a_{n})$ is a formula of IKP(E) with all free variables indicated and $s_{1},\ldots ,s_{n}$ are ${\mathrm{IRS}}_{\Omega }^{E}$ terms. The formula $A(s_{1},\ldots ,s_{n})$ is said to be $\Delta_{0}^{E}$ if $A(a_{1},\ldots ,a_{n})$ is a $\Delta_{0}^{E}$ formula of IKP(E).

An important class of formulae was isolated in [28, definition 4.3]. The ${\dot{\Sigma }}^{E}$ formulae are the smallest collection containing the $\Delta_{0}^{E}$ formulae such that A∧B, A∨B, (∀x∈t)A, (∃x∈t)A, $(\exists x\in^{a}b)A$, $(\forall x\in^{a}b)A$, ∃xA, ¬C and C→A are in ${\dot{\Sigma }}^{E}$ whenever A and B are in ${\dot{\Sigma }}^{E}$ and C is in ${\dot{\Pi }}^{E}$. The ${\dot{\Pi }}^{E}$ formulae are the smallest collection containing the $\Delta_{0}^{E}$ formulae such that A∧B, A∨B, (∀x∈t)A, (∃x∈t)A, $(\exists x\in^{a}b)A$, $(\forall x\in^{a}b)A$, ∀xA, ¬C and C→A are in ${\dot{\Pi }}^{E}$ whenever A and B are in ${\dot{\Pi }}^{E}$ and C is in ${\dot{\Sigma }}^{E}$.

For the details of the notion of operator controlled in ${\mathrm{IRS}}_{\Omega }^{E},$ we refer the reader to [28, section 4]. IKP(E) can be embedded in ${\mathrm{IRS}}_{\Omega }^{E}$, and the counterparts to theorems 3.5, 3.6, 3.7 and 3.8 ensue. We will just record the last one, which removes all inference rules $\left({\dot{\Sigma }}^{E}\text{-}Ref\right)$ from the derivation.

**Theorem 3.12. (Collapsing for ${\mathrm{IRS}}_{\Omega }^{E}$)**

*Suppose that* $\eta \in H_{\eta }$, Δ *is a set of at most one* ${\dot{\Sigma }}^{E}$-*formula and* Γ *a set of* ${\dot{\Pi }}^{E}$-*formulae. Then*

$$H_{\eta }|\frac{\alpha }{\Omega +1}\Gamma \Rightarrow \Delta \;\text{implies}\;H_{\hat{\alpha }}|\frac{\psi_{\Omega }(\hat{\alpha })}{\psi_{\Omega }(\hat{\alpha })}\Gamma^{E_{\tau }}\Rightarrow \Delta^{E_{\tau }},$$

*where* $\tau :=\psi_{\Omega }(\hat{\alpha })$, $\hat{\beta }:=\eta +\omega^{\Omega +\beta }$, *and the operators* $H_{\xi }$ *are those defined in [28] definition 2.18*.

**Proof.**

See ([28, theorem 4.13]).

### (c) Background theory for ordinal analyses

For the proof strategy of this article, it is important to ponder what background theory suffices for the task of carrying out the foregoing ordinal analyses. By the latter, we mean the embedding, cut elimination and collapsing theorems, but not the soundness theorems 2.35, 3.25 and 4.25 of [28]. If T is one of the theories IKP, IKP(P) or IKP(E) and T⊢C with C being a Σ, ${\dot{\Sigma }}^{E}$ or ${\dot{\Sigma }}^{P}$ sentence, respectively, then one can explicitly determine a natural n such that the entire ordinal analysis for this statement requires only ordinals from $B^{\Omega }(\omega_{n}(\Omega +1))$ (where $\omega_{0}(\Omega +1)=\Omega +1$ and $\omega_{k+1}(\Omega +1)=\omega^{\omega_{k}(\Omega +1)}$). Certainly, transfinite induction over the ordinal representations of $B^{\Omega }(\omega_{n}(\Omega +1))$ will be required, but actually very little beyond that. Recall that ${\mathrm{IKP}}_{R}$ arises from IKP by substituting Σ replacement for strong collection. The claim is that ${\mathrm{IKP}}_{R}$ is capacious enough.

**Theorem 3.13.**

*The proof-theoretic ordinal of* ${\mathrm{IKP}}_{R}$ *is the Bachmann–Howard ordinal. In particular, for every* n, ${\mathrm{IKP}}_{R}$ *proves transfinite induction on the ordinal presentations of* $B^{\Omega }(\omega_{n}(\Omega +1))$ *for arbitrary formulae*.

**Proof.**

This was shown in [36, theorem 4.13] to hold for the theory ${\mathrm{CZF}}_{R}^{0}$, i.e. CZF without subset collection and replacement in lieu of strong collection. Scouring its proof, it turns out that ${\mathrm{CZF}}_{R}^{0}$ can be replaced by ${\mathrm{IKP}}_{R}$. Replacement is, for instance, used in the proof of [36, theorem 2.6] on which [36, theorem 4.13] builds, but fortunately it is just an instance of Σ replacement therein.

An obstacle for formalizing ordinal analysis within ${\mathrm{IKP}}_{R}$, though, is posed by the need to formalize the notion of operator controlled derivability $H|\frac{\alpha }{\rho }\Gamma \Rightarrow A$. This notion is an example of a Σ-inductive definition (cf. [18, chapter V]). While it is no problem to formalize such definitions in the presence of Σ-collection, it does not seem to be possible with just Σ replacement at one’s disposal (see [36, section 2] for a discussion). To obviate this problem, the first observation is that instead of arbitrary operators one just needs recursive operators taking finite sets of ordinal representations as inputs and outputs. The main idea is then that we can do everything with recursive proof trees controlled by a recursive operator instead of arbitrary operator controlled derivations. A *proof-tree* controlled by a recursive operator H is a tree, with each node labelled by: a sequent, a rule of inference or the designation ‘Axiom’, two sets of formulas specifying the set of principal and minor formulas, respectively, of that inference, and two ordinals (length and cut–rank) such that the sequent is obtained from those immediately above it through the application of the specified rule of inference, additionally controlled by H. The well-foundedness of a proof-tree is then witnessed by the (first) ordinal 'tags' which are in reverse order of the tree order. Furthermore, one needs to show that all the proof-theoretic operations such as embedding, cut elimination and collapsing can be engineered via recursive functions acting on them. The upshot is that there are well-known techniques for handling infinite derivations in intuitionistic systems (even of arithmetic) via codes for recursive proof trees or even primitive recursive ones (see, for instance [37–41]), and that this treatment is deployable in ${\mathrm{IKP}}_{R}$.

## 4. Partial conservativity over systems based on replacement

The ordinal analyses of IKP, IKP(E) and IKP(P) of [28] can be used to obtain conservativity results over the counterparts with replacement in lieu of collection. We will use the acronyms ${\mathrm{IKP}}_{R}$, ${\mathrm{IKP}}_{R}(P)$ and ${\mathrm{IKP}}_{R}(E)$ for the theories with Σ replacement, $\Sigma^{E}$ replacement and $\Sigma^{P}$ replacement instead of Σ collection, $\Sigma^{E}$ collection and $\Sigma^{P}$ collection, respectively.

**Theorem 4.1.**

(i) $\mathrm{IKP}+\dot{\Sigma }\text{-reflection}$ *is conservative over* ${\mathrm{IKP}}_{R}$ *for* $\dot{\Sigma }$-*sentences*.
(ii) $\mathrm{IKP}(E)+{\dot{\Sigma }}^{E}\text{-reflection}$ *is conservative over* ${\mathrm{IKP}}_{R}(E)$ *for* ${\dot{\Sigma }}^{E}$-*sentences*.
(iii) $\mathrm{IKP}(P)+{\dot{\Sigma }}^{P}\text{-reflection}$ *is conservative over* ${\mathrm{IKP}}_{R}(P)$ *for* ${\dot{\Sigma }}^{P}$-*sentences*.

**Proof.**

Let us start with (iii). So suppose that $\mathrm{IKP}(P)+{\dot{\Sigma }}^{P}\text{-reflection}$ proves a ${\dot{\Sigma }}^{P}$-sentence A. The length of this finite deduction determines a number n such that the entire ordinal analysis for this deduction, comprising the embedding theorems 3.5, theorem 3.6 and the collapsing theorem 3.8, solely uses ordinals from the set $B^{\Omega }(\omega_{n}(\Omega +1))$, where $\omega_{0}(\Omega +1)=\Omega +1$ and $\omega_{k+1}(\Omega +1)=\omega^{\omega_{k}(\Omega +1)}$. Moreover, it follows from theorem 3.13 that transfinite induction along the ordinals of $B^{\Omega }(\omega_{n}(\Omega +1))$ is provable in ${\mathrm{IKP}}_{R}$ for arbitrary formulae. As argued in §3c, we can determine a recursive operator H, a recursive proof-tree and ordinals α,ρ<Ω with $\alpha ,\rho \in B^{\Omega }(\omega_{n}(\Omega +1))$ such that $H|\frac{\alpha }{\rho }\Gamma \Rightarrow A$, and, moreover, we gain this insight in ${\mathrm{IKP}}_{R}$.

Switching to ${\mathrm{IKP}}_{R}(P)$, we aim to conclude that A is also true. Temporarily, let $VAR_{P}$ be the set of variables with labels in $B^{\Omega }(\omega_{n}(\Omega +1))$. Moreover, we only consider ${\mathrm{IRS}}_{\Omega }^{P}$-terms created from ordinals in $B^{\Omega }(\omega_{n}(\Omega +1))$. Let $V_{\alpha }$ be obtained by iterating the powerset operation α-times for $\alpha \in B^{\Omega }(\omega_{n}(\Omega +1))\cap \Omega$, i.e. for ξ,λ<α, let

V0:=∅,AAAAVξ+1:={X|X⊆Vξ},AAAAVλ:=⋃β<λVβfor λ a limit.

This hierarchy is definable in ${\mathrm{IKP}}_{R}(P)$ using $\Sigma^{P}$ replacement and powerset since one has transfinite induction over $B^{\Omega }(\omega_{n}(\Omega +1))\cap \Omega$ (for any formula) and the case distinctions (as to zero, successor and limit ordinal (representation)) are decidable.

We then only consider variable assignments $v:VAR_{P}⟶V_{\psi_{\Omega }(\omega_{n}(\Omega +1))}$, of course satisfying $v(a_{i}^{\alpha })\in V_{\alpha +1}$ for each i. The assignment v canonically propagates to all terms via

$$v(V_{\alpha })=V_{\alpha }$$

and

$$v(\{x\in V_{\alpha }|F(x,s_{1},\ldots ,s_{n})\})=\{x\in V_{\alpha }|F(x,v(s_{1}),\ldots ,v(s_{n}))\}.$$

Moreover, it can be seen that $v(s)\in V_{|\;s\;|+1}$, and thus, $v(s)\in V_{\psi_{\Omega }((\omega_{n}(\Omega +1))}$.

We then obtain the following soundness result for ${\mathrm{IRS}}_{\Omega }^{P}$ as in [28, theorem 3.25]: Suppose $\Gamma [s_{1},\ldots ,s_{n}]$ is a finite set of ${\dot{\Pi }}^{P}$ formulae with max{rk(A)|A∈Γ}≤Ω and $\Delta [s_{1},\ldots ,s_{n}]$ a set containing at most one ${\dot{\Sigma }}^{P}$ formula such that

$$H|\frac{\alpha }{\rho }\Gamma [\bar{s}]\Rightarrow \Delta [\bar{s}]\;\text{for some operator }H\text{ and some }\alpha ,\rho \in B^{\Omega }(\omega_{n}(\Omega +1))\cap \Omega .$$

Then, for any assignment v going to $V_{\psi_{\Omega }(\omega_{n}(\Omega +1))}$,

$$V_{\psi_{\Omega }((\omega_{n}(\Omega +1))}⊨⋀\Gamma [v(s_{1}),\ldots ,v(s_{n})]\to ⋁\Delta [v(s_{1}),\ldots ,v(s_{n})].$$

Here, ⋀Γ and ⋁Δ stand for the conjunction of formulae in Γ and the disjunction of formulae in Δ, respectively (by convention ⋀∅=⊤ and ⋁∅=⊥). For a more detailed proof, see [28, 3.25].

By soundness, we therefore have $V_{\psi_{\Omega }((\omega_{n}(\Omega +1))}⊨A$ for the assignment $v_{s}$, which interprets any variable by ∅. As a result, A is provable in ${\mathrm{IKP}}_{R}(P)$.

We now turn to the proof of (ii), which is to a large extent similar to that of (iii). Of course, it is based on the much more complicated ordinal analysis of IKP(E) from [28] section 4. Analogously to ${\mathrm{IRS}}_{\Omega }^{P}$, one proves a soundness theorem for certain ${\mathrm{IRS}}_{\Omega }^{E}$ derivable sequents. Again we consider only variable assignments

$$v:VAR_{E}⟶E_{\psi_{\Omega }(\omega_{n}(\Omega +1))},$$

such that $v(a_{i}^{\alpha })\in E_{\alpha +1}$ for all i<ω and ordinals α. Here, $E_{\beta }$ refers to the E-hierarchy of definition 3.10. Again, such an assignment propagates to all ${\mathrm{IRS}}_{\Omega }^{E}$ terms by letting

$$v(E_{\alpha })=E_{\alpha }$$

and

$$v([x\in t|F(x,s_{1},\ldots ,s_{n})])=\{x\in v(t)|F(x,v(s_{1}),\ldots ,v(s_{n}))\}.$$

As mentioned earlier, the crucial difference between here and the case of ${\mathrm{IRS}}_{\Omega }^{P}$ is that for a given term t, it is no longer possible to describe the location of v(t) within the E-hierarchy solely by inspecting its syntactic structure, albeit it is possible to place an upper bound on that location using the following function:

$$\begin{array}{rl} m(E_{\alpha }) & \;:=\alpha \\ m(a_{i}^{\alpha }) & \;:=\alpha \\ \mathrm{and}\;\;\;\;m([x\in t|F(x,s_{1},\ldots ,s_{n})]) & \;:=\text{max}(m(t),m(s_{1}),\ldots ,m(s_{n}))+1. \end{array}$$

We then have that $v(s)\in E_{m(s)+1}$ for any s, though in general m(s) only determines an upper bound on a term’s position in the E-hierarchy. According to [28, theorem 4.25], one obtains the following soundness result for ${\mathrm{IRS}}_{\Omega }^{E}$. Let $\Gamma [s_{1},\ldots ,s_{n}]$ be a finite set of ${\dot{\Pi }}^{E}$ formulae with max{rk(A)|A∈Γ}≤Ω and $\Delta [s_{1},\ldots ,s_{n}]$ be a set containing at most one ${\dot{\Sigma }}^{E}$ formula such that

$$H|\frac{\alpha }{\rho }\Gamma [\bar{s}]\Rightarrow \Delta [\bar{s}]\;\text{for some operator }H\text{ and some }\alpha ,\rho \in B^{\Omega }(\omega_{n}(\Omega +1))\cap \Omega .$$

Then, given any assignment v going into $E_{\psi_{\Omega }(\omega_{n}(\Omega +1))}$,

$$E_{\psi_{\Omega }(\omega_{n}(\Omega +1))}⊨⋀\Gamma [v(s_{1}),\ldots ,v(s_{n})]\to ⋁\Delta [v(s_{1}),\ldots ,v(s_{n})].$$

Finally, for (i), we use the constructible hierarchy defined by transfinite recursion along the ordinals of $B^{\Omega }(\omega_{n}(\Omega +1))\cap \Omega$.

$$\begin{array}{rl} L_{0} & \;:=\emptyset \\ L_{\xi +1} & \;:=\{X|X\text{ is definable over }\langle L_{\xi },\in \rangle \text{ with parameters}\}\\ \mathrm{and}\;\;\;\;L_{\lambda } & \;:=\underset{\beta <\lambda }{⋃}L_{\beta }\;\text{for }\lambda \text{ a limit.} \end{array}$$

This hierarchy is definable in ${\mathrm{IKP}}_{R}$ using just $\Sigma^{P}$ replacement rather than Σ collection (for details, see [42]).

Noting that they do not contain free variables, terms t of ${\mathrm{IRS}}_{\Omega }$ (see [28, subsection 2.3]), are assigned to sets v(t) in the obvious way, namely,

$$v(L_{\alpha })=L_{\alpha }$$

and

$$v([x\in t|F{\left(x,s_{1},\ldots ,s_{n}\right)}^{L_{\alpha }}])=\{x\in v(t)|F(x,v(s_{1}),\ldots ,v{\left(s_{n})\right)}^{L_{\alpha }}\}.$$

As a result, one gets a soundness theorem for ${\mathrm{IRS}}_{\Omega }$ in the same way as for ${\mathrm{IRS}}_{\Omega }^{P}$, yielding (i).

## 5. Existence property for theories with replacement

Let ${\mathrm{CZF}}_{R}^{-}$, ${\mathrm{CZF}}_{E,R}$ and ${\mathrm{CZF}}_{P,R}$ be the counterparts theories obtained from of ${\mathrm{CZF}}^{-}$, ${\mathrm{CZF}}_{E}$ and ${\mathrm{CZF}}_{P}$, respectively, by having the replacement scheme instead of the strong collection scheme.

From theorems 4.1 and 2.12, it follows that ${\mathrm{CZF}}^{-}$, ${\mathrm{CZF}}_{P}$ and ${\mathrm{CZF}}_{E}$ have the EP if ${\mathrm{CZF}}_{R}^{-}$, ${\mathrm{CZF}}_{P,R}$ and ${\mathrm{CZF}}_{E,R}$, respectively, have this property. Techniques to establish the EP for intuitionistic set theories based on replacement rather than collection are available. Friedman [2] introduced a modification of Kleene’s slashing technology and used it to prove the EP for various systems of type theory and higher-order arithmetic. These results were extended to versions of intuitionistic Zermelo–Fraenkel set theory with replacement by Friedman and Myhill in [3] and to constructive set theories by Myhill [4].

The technology can also be deployed in cases such as the theories ${\mathrm{CZF}}_{P,R}$ and ${\mathrm{CZF}}_{E,R}$. Owing to it being well-documented in the research literature (especially [3,4], and also in book form in [1], IX) and due to page restrictions, I shall just sketch the main steps.^6^

**Definition 5.1.**

Let L be a first-order language comprising the language of set theory, $S_{L}$ its set of sentences, $A_{L}$ its atomic sentences and $T_{L}$ its set of closed terms. For T a set of L-sentences, let Thm(T) be $\left\{A\in S_{L}|T⊢A\right\}$. Let $M\subseteq A_{L}$ be a non-empty set of atoms and $P\subseteq S_{L}$ such that Thm(T)⊆P and P is closed under modus ponens, i.e. A∈P and A→D∈P yields D∈P.

Friedman realizability, R(T,M,P), is the unique set $F\subseteq S_{L}$ such that the following conditions are met.

$$\begin{array}{rl} & G\in F\Leftrightarrow G\in M,\text{ for }G\in A_{L},\\ & \;⊥\notin F\\ & A\wedge D\in F\Leftrightarrow A\in F\text{ and }D\in F,\\ & A\vee D\in F\Leftrightarrow A\in P\cap F\text{ or }D\in P\cap F,\\ & A\to D\in F\Leftrightarrow A\in P\cap F\text{ implies }D\in F,\\ & \forall x\;B(x)\in F\Leftrightarrow B(t)\in F\text{ for all }t\in T_{L},\\ \mathrm{and}\;\;\;\; & \exists x\;B(x)\in F\Leftrightarrow B(t)\in P\cap F\text{ for some }t\in T_{L}. \end{array}$$

We say that *T realizes A relative to M and P* if A∈R(T,M,P).

The fundamental property of R is given by the following.

**Theorem 5.2. (Friedman)**

T⊆R(T,M,P) *implies that* Thm(T)⊆R(T,M,P).

**Proof.**

[2], theorem 2.1.

Let us give an outline as to how it can be shown that ${\mathrm{IKP}}_{R}$, ${\mathrm{IKP}}_{R}(P)$, ${\mathrm{IKP}}_{R}(E)$, ${\mathrm{CZF}}_{R}^{-}$, ${\mathrm{CZF}}_{P,R}$ and ${\mathrm{CZF}}_{E,R}$ (and similar theories) possess the EP. Let T be any of these theories. T will just serve the purpose of a generic example to which this technology applies. Suppose T⊢∃u A(u), where the formula has no free variables. In a first step, one builds a conservative extension ${\text{T}}^{+}$ of T such that we have ${\text{T}}^{+}⊢A(t)$ for some term t of the enriched language. Translating back to the original language will give us the result. In the light of theorem 5.2, we only need to produce such a term t for each set existence axiom of T. As it suffices to show that there is a formula G(x) such that

T⊢∃u [A(u) ∧ ∀x (x∈u↔G(x))],

the idea is to introduce comprehension terms.

**Definition 5.3.**

For each formula G(x) with at most x free such that T⊢∃y ∀x (x∈y↔G(x)), we introduce a comprehension term [x∣G(x)]. Two comprehension terms s,t are viewed as equivalent if their defining formulae are provably equivalent in T, and we write s∼t to convey this. We extend the language of set theory by a new constant $c_{t}$ for each comprehension term t≡[x∣G(x)], and then write $G_{t}(x)$ for G(x). Let $L^{\prime }$ be this new language and $T^{\prime }$ be the theory in this new language consisting of the axioms of T along with

$$\forall x\;(x\in c_{t}↔G_{t}(x)),$$

for each comprehension term t.

**Proposition 5.4.**

$T^{\prime }$ *is a conservative extension of* T.

**Proof.**

This is achieved by simply replacing every atomic $t\in t^{\prime }$, with $t,t^{\prime }$ comprehension terms, by

$$\exists u\exists v\;[\forall x\;(x\in u↔G_{t^{\prime }}(x))\;\wedge \;\forall x\;(x\in v↔G_{t}(x))\;\wedge \;v\in u].$$

To arrive at the theory ${\text{T}}^{+},$ we need an even larger class of set-indexed constants, $C^{+}$.

**Definition 5.5.**

$C^{+}$ is defined via the following inductive definition:^7^

If c is a constant of $L^{\prime }$ and $X\subseteq \{c_{Y}\in C^{+}|T^{\prime }⊢c\in b\}$, then $b_{X}\in C^{+}$.

For $d\equiv b_{X}\in C^{+}$, we write $d^{-}$ for b, and $d^{+}$ for X.

Let $L^{+}$ be the extension of $L^{\prime }$ via the new set-indexed constants. Let ${\text{T}}^{+}$ be the theory in the language $L^{\prime }$ with the axioms of ${\mathrm{CZF}}_{P,R}^{\prime }$ augmented by the axioms

$$\forall x\;[x\in d↔A_{t}(x)],$$

where $d^{-}\;\equiv \;c_{t}$ for a comprehension term t.

**Proposition 5.6.**

${\text{T}}^{+}$ *is a conservative extension of* T.

**Proof.**

The proof idea is for a formula A of $L^{+}$ to replace each occurrence of $d\in C^{+}$ by $d^{-}$.

Finally, we are in a position to make use of theorem 5.2. In the latter let T be T and P be $Thm({\text{T}}^{+})$ and M be the set of atomic sentences e∈d, where e belongs to $d^{+}$. The pivotal move is to show that for each set existence axiom

∃y∀x [x∈y↔A(x)],

of ${\text{T}}^{+}$, there is a set X so that

e∈X ⇔ A(e)∈P∩F,

where F=R(T,M,P). As a result, for b∼[x∣A(x)], we will have ∃y∀x [x∈y↔A(x)]∈P∩F, and hence, by theorem 5.2, $Thm(T^{\prime })\subseteq F$.

Accordingly, if T⊢∃u H(u), then ∃u H(u)∈F, so that there is a $s\in C^{+}$ with ${\text{T}}^{+}⊢H(d)$. Consequently, $T^{\prime }⊢H(d^{-})$. As $d^{-}$ is of the form $c_{t}$ for a comprehension term t, we arrive at

$$T⊢\exists u\;[H(u)\wedge \forall x(x\in u↔A_{t}(x))].$$

**Theorem 5.7.**

${\mathrm{IKP}}_{R}$, ${\mathrm{IKP}}_{R}(E)$, ${\mathrm{IKP}}_{R}(P)$, ${\mathrm{CZF}}_{R}^{-}$, ${\mathrm{CZF}}_{E,R}$ *and* ${\mathrm{CZF}}_{P,R}$ *have the EP.*

The aforementioned proof works for a wide variety of theories whose set-existence axioms are explicit, i.e. if they define the denizens of the set being asserted to exist in the form

∀u [D(u )→∃y∀x (x∈y↔G(x,u ))].

In [3, section 6], it is shown in detail that axioms of the aforementioned form are always realizable.

## 6. Conclusion

Preparations are finished now, and we can show that various theories with collection axioms have the EP.

**Theorem 6.1.**

IKP, IKP(E), IKP(P), ${\mathrm{CZF}}^{-}$, ${\mathrm{CZF}}_{E}$ *and* ${\mathrm{CZF}}_{P}$ *have the* EP.

**Proof.**

As an example we show this for ${\mathrm{CZF}}_{E}$. By theorem 2.12 (ii), it suffices to show that IKP(E) has the EP for $\Sigma^{E}$ formulae. So assume IKP(E)⊢∃xB(x) for a closed $\Sigma^{E}$ formula. Theorem 4.1(ii) yields that ${\mathrm{IKP}}_{R}(E)⊢\exists xB(x)$, and hence, invoking theorem 5.7, there is a formula C(x) (with at most x free) such that ${\mathrm{IKP}}_{R}(E)⊢\exists !x\;[C(x)\;\wedge \;B(x)]$. In consequence, IKP(E)⊢∃!x [C(x) ∧ B(x)].

The proofs for the other theories follow the same pattern.

## Data Availability

This article has no additional data.

## Footnotes

1 If one assumes a choice principle, called the presentation axiom, exponentiation yields subset collection (cf. [15], 10.3.3).

2 The statement that for any two sets the class of multi-valued functions between them is a set is too strong as it is equivalent to powerset (cf. [15], 5.1.6).

3 Burr ([16], corollary 5.12) and Diller ([17], proposition 4.4) showed that higher type versions of constructive set theory, but without subset collection and exponentiation, enjoy weak forms of the term existence property.

4 ([12], theorem 3.10) does not state this for IKP, IKP(E) and IKP(P), but it is easily checked that theorems 3.7–3.9 of [12] also work for IKP, IKP(E) and IKP(P), respectively, from which this follows.

5 Note that the ${\dot{\Sigma }}^{P}$-formulae comprise the $\Sigma^{P}$-formulae of definition 2.7. In the terminology of [28], however, the $\Sigma^{P}$-formulae are identical to the ${\dot{\Sigma }}^{P}$-formulae, but this clashes with the terminology of [12], hence the dot.

6 In 2012, I attended a talk by Ali Lloyd who was a PhD student of Peter Aczel back then. It was about ascertaining the EP for ${\mathrm{CZF}}_{E,R}$, with a discussion of the metatheory required for such a proof. The approach was also based on Friedman realizability as in this section. Moreover, he mentioned in the talk that Peter Aczel had shown the EP for ${\mathrm{CZF}}_{E,R}$ with a proof being formalizable in ${\mathrm{IZF}}_{R}$. Alas, I was unsuccessful finding any traces of this on the worldwide net.

7 Notably, this kind of definition can be formalized in ${\mathrm{CZF}}^{-}$ as a class inductive definition.
